# Determinant Factors of Sexual and Reproductive Health Service Utilization among In-School Adolescents with Disability in Jimma Zone, Southwest Ethiopia

**DOI:** 10.1155/2022/5945921

**Published:** 2022-08-18

**Authors:** Tujuba Diribsa, Diriba Wakjira, Gamechu Atomsa, Tsiyon Mekoya, Fedhesa Mamo, Bekana Fekecha, Mosisa Ebisa, Girma Teferi

**Affiliations:** ^1^Department of Population and Family Health, Jimma University, Jimma, Ethiopia; ^2^Department of Midwifery, Jimma University, Jimma, Ethiopia; ^3^Department of Nursing, Jimma University, Jimma, Ethiopia; ^4^Department of Nursing, Wolkite University, Wolkite, Ethiopia

## Abstract

**Introduction:**

Adolescents with disability are often presumed erroneously to be sexually inactive. Though they have the same need for sexual and reproductive health (SRH) services as nondisabled people, they are often overlooked by sexual and reproductive health programs, interventions, and studies.

**Objective:**

To assess determinant factors of sexual and reproductive health service utilization among in-school adolescents with disability in Jimma zone, southwest Ethiopia.

**Method:**

Institution-based cross-sectional study design was employed among in-school adolescents with disability in Jimma zone, Ethiopia, from September 21 to November 30, 2021. A total of 454 participants were included in the study by using the multistage sampling technique. Data were collected by using a structured questionnaire through face-to-face interviews. Data were entered into Epi-data version 4.2 and analyzed by using SPSS version 23. Bivariate and multivariable logistic regression analyses at a 95% confidence interval were performed, and a *P* value < 0.05 was considered statistically significant.

**Result:**

454 study participants were included in this study with a respondent rate of 97.4%. Only 38 (8.4%, 95% CI: 5.7-10.8%) of in-school adolescents utilized SRH information and education service. The majority (265, 49%) of adolescents with disability knew family planning as sexual and reproductive health services which were followed by voluntary counselling and testing for HIV/AIDS (116, 21.4%). Seventy-eight (17.2%, 95% CI: 13.7-20.5%) of in-school adolescents with disability visited nearby health facilities for VCT services. Male sex (AOR = 2.32, 95% CI: 1.18-4.57), favourable attitude (AOR = 3.11, 95% CI: 1.59-6.07), and history of sexual intercourse (AOR = 5.34, 95% CI: 2.05–13.92) were significantly associated with SRH service utilization.

**Conclusion:**

The overall sexual and reproductive health service utilization of in-school adolescents with disability was low when compared with other studies. Physical impairment, male sex, ever had sexual intercourse, good knowledge, and favourable attitudes were determinant factors of SRH service utilization among in-school adolescents with disabilities. So, it is recommended that the Jimma zone administration, government, and NGOs should give attention to SRH services.

## 1. Introduction

Globally, there are more than one billion people with disabilities and the average global disability prevalence rate is estimated to be 15.6%. Around 180 to 220 million young people live with disability in the world; nearly 80% live in developing countries [1]. There are an estimated 15 million people with disabilities in Ethiopia, comprising physical and intellectual disability and hearing and visual impairment [2]. People with disability are the most disadvantaged section of society; they are neglected in their families and different social services. Adolescents with disability are among the poorest and most marginalized groups of the world's young people [1].

Most health promotion and prevention services often do not target people with disabilities in terms of communication and provision. Because of this reason, health disparities and inequality are seen in the areas of health outcomes, preventive screening programs, and health-promoting behaviors [3]. People with disability are sexually active as others and have similar needs for SRH services [4]. However, they are often overlooked by SRH programs and health services [5, 6].

A study conducted in urban areas of Sierra Leone indicated that people with disabilities are two times less likely to access health services including reproductive health services as compared to nondisabled people [7]. Similarly, a study conducted in Ghana showed that 87% of the young people with disability had ever faced a barrier in their quest to access SRH services [8].

Local evidence from a study conducted in Addis Ababa also depicted that 64.6% of young people with disability (YPWD) have heard about SRH service, but only 26.1% had utilized SRH service, 76.5% of YPWD indicated that they would undergo VCT if the service would be available, whereas 23.5% had no such intention, and 29.6% of the respondents preferred to discuss topics of sexuality and reproductive health with health professionals [9]. Similar evidence also showed that the magnitude of unintended pregnancy was 62.5% among young disabled females who had ever been pregnant, 50% of them had a history of abortion, and 87.5% of those abortions were induced type [10].

Young people with disability face a multitude of challenges in accessing sexual and reproductive health (SRH) services. Evidence from different studies showed that negative attitudes of service providers, long waiting at health facilities, distant health facilities, unfriendly physical structures, parent's disproval, and lack of information on the existence of the services are the major challenges of YPWD in assessing SRH service. And also, most health care professionals have no disability awareness and are unable to communicate in sign language, consequently unable to address their issues [9, 11–13].

Sustainable Development Goal (SDG) 3 target 3.7, which calls for universal access to sexual and reproductive health (SRH) services [14]. Furthermore, article 25 of the Convention on the Right of People with Disability (CRPD) states that persons with disabilities have the right to the enjoyment of the highest attainable standard of health without discrimination based on disability, including in the area of SRH [6].

Ethiopian national adolescent and youth health strategy (2016-2020) recognized the need to give attention to the SRH of adolescents and youth with disabilities [15]. Since the exact nature of SRH needs and utilization of adolescents with disabilities is unknown, this strategy is unlikely to be successful in addressing existing inequities in access, quality of services, and outcomes for adolescents with disabilities.

In general, little research has been conducted on the SRH of people with disabilities worldwide, and there are very few disability-specific studies on SRH service utilization of adolescents. Particularly in Ethiopia, to the knowledge of investigators, there is no study conducted on SRH service utilization among adolescents with disability. So, for evidence-based and context-specific interventions that meet the SRH needs of adolescents with disability, it is vital to have a comprehensive understanding of SRH utilization services. To fill the gap, this study was aimed at assessing sexual and reproductive health service utilization and its determinant factors among in-school adolescents with disability in Jimma zone, southwest Ethiopia, 2021.

## 2. Method and Materials

### 2.1. Study Area, design and Period

The study was conducted in primary and secondary schools found in the Jimma zone. Jimma zone is located 346 km in the southwest direction from the capital city of Ethiopia, Addis Ababa. Jimma zone is one of the largest zones of the Oromia region. The zone has 21 woredas and two city administrations. Jimma town is the capital city of the zone. According to the report from the Jimma zone educational bureau in the academic year of 2021, there were 1127 primary schools (grades 1-8) and 92 secondary schools (grades 9-12), and a total of 2792 students with disabilities were enrolled in primary and secondary schools in 21 woredas of Jimma zone and two city administrations. The study was carried out from September 21 to November 30, 2021.

#### 2.1.1. Study Design

Institution based cross-sectional study design was conducted.

#### 2.1.2. Source Population

All adolescents with disability enrolled in primary and secondary schools found in the Jimma zone were the source population.

#### 2.1.3. Study Population

All randomly selected adolescents with disabilities, enrolled in primary and secondary schools of Jimma Zone, in the academic year of 2021 were the study population.

### 2.2. Inclusion and Exclusion Criteria

Adolescents with disability enrolled in primary and secondary schools of Jimma zone in the academic year of 2021 were included, and those critically ill and unable to respond and unwilling to participate in the study were excluded.

### 2.3. Sample Size Estimation

The sample size was determined for the specific objective of this study, and the largest sample size which applies to all objectives was considered. The sample size was calculated using a single population proportion formula.

(1) $$\begin{array}{c} n=\frac{{\left(z_{1-\alpha /2}\right)}^{2}\times p\;\left(1-p\right)\;}{d^{2}}, \end{array}$$

where *n* is the sample size, *Z*_1−*α*/2_ is the confidence level corresponding to 95%CI = 1.96, *P* is the previous prevalence rate, and *d* = 0.05 is the margin of the sampling error to be tolerated. Based on the above assumption, the following table summarizes the sample size for different major variables of these studies. The sample size for associated factors was determined by Epi-Info 7 software.

From the below table, the large sample size was 444. By adding 5% of the nonresponse rate, the final sample size required for this study was 466 (Table 1).

### 2.4. Sampling Technique and Procedures

A multistage sampling technique was used. Before the study, preliminary data (name of woreda, name of the school, a form of disability, grade/class, serial number/ID no, sex, and age) of the adolescents with disability attending primary and secondary schools in the Jimma zone was obtained from all woredas found in Jimma zone. Then, 7 woredas (i.e., about 30% of all woredas) and 1 city administration were randomly selected. Based on the number of schools, again 30% of them were selected by lottery method from each selected woreda and city administration. Finally, the adolescents with disability were selected by a simple random sampling technique from each selected school by using student lists as a sampling frame.

### 2.5. Data Collection Tools and Techniques

A structured questionnaire was adapted after reviewing relevant literature. The questionnaire was prepared in the English language. It was translated to the local language (Afan Oromo and Amharic) and then back to English by a language expert to check its consistency. The data were collected through face-to-face interviews of the study participants. Female data collectors were assigned to collect data from females and male data collectors to collect data from males. To collect data from participants with hearing impairments, a sign language translator was assigned with data collectors. During data collection, COVID-19 preventive practices like wearing face masks were strictly applied by data collectors. Materials like face masks and sanitizer were arranged and adequately distributed for data collectors and supervisors.

### 2.6. Study Variables

#### 2.6.1. Dependent Variable

The dependent variable is SRH service utilization.

#### 2.6.2. Independent Variable

The following are the independent variables.

- Sociodemographic characteristics: age, residence, sex, educational level, marital status, living condition, source of income
- Disability-related factors: form of disabilities and time of disability
- Individual attribute factors: history of sexual intercourse, discussion on SRH, SRH knowledge, self-risk perception, and attitude towards SRH
- Health facility service-related factors: fear of service providers' confidentiality and privacy, perception of the friendliness of SRH service, knowing where to get SRH service, perceived accessibility, and need an assistant to get SRH service

### 2.7. Operational Definition

Adolescent: persons between the ages of 10 and 19 years old [15].

An adolescent with disability: in this study, adolescents with physical, visual, hearing, and mental impairments and reported by schools as special needy students [16].

Knowledge about sexual and reproductive health services: participants were asked knowledge questions. Those who score the mean and above were categorized as having good knowledge, and those who scored below the mean were considered as having poor knowledge [17].

Attitude: attitudes on the SRH issue were assessed by attitudinal statements. Based on three-point Likert scales for each statement, participants could choose between three possible response categories: “agree,” “neutral,” or “disagree.” Then, overall score was calculated; participants who score mean and above were considered as having a favourable attitude and below the mean score was an unfavourable attitude [9].

Self-risk perception on HIV/AIDS/STI: adolescents with disability were asked to rate their self-risk toward susceptibility to HIV/STI as high, low, and no risk at all [16].

Contraceptive service utilization: adolescents who used any of the modern birth controlling methods (contraceptives) in the past 12 months [18].

SRH information and education service utilization: adolescents who received information and education regarding sexual and reproductive health issues from health workers working in any of the service providing points within the past 12 months [18].

STI diagnosis and treatment service utilization: adolescents who obtained STI diagnosis and treatment service in the past 12 months [18].

VCT service utilization: in this study, adolescents who received HIV counselling and testing service in the past 12 months [16].

### 2.8. Data Management and Statistical Analysis

The questionnaire was checked for completeness, cleaned, coded, and then entered into Epi-data statistical software version 4.2 and exported to Statistical Package for the Social Sciences (SPSS) Windows version 23 for analysis. Frequencies, proportions, and summary statistics were used to describe the study population about relevant variables and presented using texts and tables. The bivariate analysis was employed to identify candidate variables for multivariable analysis. Then, the variables with *P* values of less than 0.25 were taken to multivariable logistic regression for controlling the possible effects of confounders. Finally, variables that had a *P* value < 0.05 were described as statistically significant, and the adjusted odds ratio (AOR) with 95% CI was determined to see the strength of the associations. Hosmer and Lemeshow test was checked, and the final model was fitted (*P* value = 0.218).

### 2.9. Quality Assurance

Quality of data was assured through careful design, translation, and back retranslation of the questionnaire. Before actual data collection, a pretest was conducted on 5% of the sample size at nonselected schools to ensure clarity, logical sequence, skip pattern of the questionnaire, and internal reliability. Internal consistency of the tools was checked by Cronbach alpha, and the amendment was done based on the findings of the pretest. Sign language translators were recruited to collect data from participants with hearing impairments. Training for two days was given to data collectors and supervisors before the actual data collection. The overall supervision was carried out by the principal investigator. During data collection, the questionnaire was reviewed and checked for completeness and consistency by supervisors every day and necessary feedback was offered to data collectors the next morning.

### 2.10. Ethics Consideration

Ethical clearance was obtained from the Institutional Review Board (IRB) of Jimma University Institute of Health. A letter of cooperation was written to Jimma Zone Educational Bureau. Permission to undertake the study was secured from all relevant authorities in the zone. Study participants were provided comprehensive information about the nature, objectives, benefits, and potential risks of the study, and participants' right to refuse and withdraw the participation in the study was assured. Written informed consent was obtained from all study participants before the interview. For those adolescents below the age of 18 years, written informed consent was obtained from the adolescent's parents/guardians. Participation was completely voluntary, and confidentiality of the information was guaranteed by a secret code and kept anonymously. Privacy and confidentiality of collected information were ensured. Adolescents with disability who required sexual and reproductive services and where to get the services were linked with surrounding health extension workers or health facilities.

## 3. Results

### 3.1. Sociodemographic Characteristics of Study Participants

The response rate for this study was 97.4%. Two hundred and fifty-eight (56.8%) of the study participants were male while 196 (43.2%) were female. The majority (70.5%) of respondents were Muslim religious followers. The majority of in-school adolescents with disability were in grades 1-4 which accounts for 48.2% and was found in age groups of 15-19 years (260, 57.3%) with a mean age of 14.99 years (±2.84) (Table 2).

### 3.2. Form and Time of Disability

The majority (144, 31.7%) of in-school adolescents with disability had a mental impairment. More than half (242, 53.3%) of disability occurred from the time of birth and followed by early childhood disability which accounted for 152 (33.5%) (Table 3).

### 3.3. Awareness and Attitude toward Sexual and Reproductive Health Services

The majority (265, 49%) of in-school adolescents with disability were aware of family planning as sexual and reproductive health services which were followed by voluntary counselling and testing for HIV/AIDS (116, 21.4%). Their primary source of information was radio and television which account for 192 (54.1%). More than half of them knew where the SRH services might be found, and 224 (49.3%) had favourable attitude about the services. Forty-one of them had visited commercial sex workers, and seventeen had multiple sexual partners (Table 4).

### 3.4. Sexual and Reproductive Health Services

Only seventy-eight (17.2%, 95% CI: 13.7-20.5%) of the in-school adolescents with disability visited a nearby health facility for VCT service, and 38 (8.4%, 95% CI: 5.7-10.8%) of them utilized SRH information and education services. Three hundred twenty (70.5%) of study participants believed that the existing SRH services are not disability friendly, and 386 (85%) of them think that the available health care facilities are not physically accessible (Table 5).

### 3.5. Sexual and Reproductive Health Status

Forty-eight (10.6%) of in-school adolescents with disability had a history of sexual intercourse and two of them being raped. The majority of respondents had used the injectable contraceptive method, and four of the study participants had a history of pregnancy (Table 6).

### 3.6. Determinant Factors of Sexual and Reproductive Health Services

Male in-school adolescents with disability were two times more likely to utilize SRH services when compared with female adolescents with disability (AOR = 2.32, 95% CI: 1.18-4.57). Again, those with a history of sexual intercourse were five times more likely to utilize SRH service than those without a history of sexual intercourse (AOR = 5.34, 95% CI: 2.05–13.92). Respondents who had a favourable attitude toward the services were three times more likely to utilize the SRH services when compared to those with unfavourable attitude (AOR = 3.11, 95% CI: 1.59-6.07) (Table 7).

## 4. Discussion

This study revealed that sexual and reproductive health information and education service utilization among in-school adolescents with disability was only 8.4% (5.7-10.8%), which was lower than the study conducted among students in public universities in Addis Ababa, Ethiopia, which was 14.87% among those with disabilities and 31.97% among those without disabilities [19]. The discrepancy may be due to a study conducted in Addis Ababa was among university students close for information and also included both those with and without disabilities.

Approximately around 17.2% (13.7-20.5%) of the in-school adolescents with disability utilized voluntary counselling and testing for HIV services. This finding was similar to a study conducted in Ghana among in-school young people with disabilities which said that about 17.1% of students reported having ever tested for HIV [20] but lower than the study conducted in Addis Ababa, Ethiopia, which was 46% of people with disabilities (PWD) had been tested for HIV [16]. The difference may be due to this study conducted among in-school adolescents with disability but that one was among people with disabilities.

About twenty-six (5.7%) of the in-school adolescents with disability utilized contraceptive services; this finding was lower than the study conducted in Osun State, Nigeria, which stated that 34% of physically challenged in-school adolescents had used a modern contraceptive method [21]. The discrepancy may be due to this study included all forms of disability. Again, lower than a study conducted among students in public universities in Addis Ababa, Ethiopia, which stated that 21.93% of students used modern contraceptive methods [19]. The discrepancy may be due to the study population difference.

In this study, male in-school adolescents with disability were two times more likely to utilize sexual and reproductive services when compared with female adolescents with disability. But, from the finding of a study conducted in Ghana, there was no difference between male and female adolescents with disability in the utilization of SRH services [20]. The discrepancy may be due to socioeconomics and cultural difference.

A finding of this study shows that in-school adolescents with disability who had good knowledge were more likely to utilize SRH services than those with poor knowledge. This finding was consistent with the study conducted in Arba Minch town, Ethiopia [22].

## 5. Conclusion

The overall sexual and reproductive health service utilization of in-school adolescents with disability was low when compared with other studies. Physical impairment, male sex, ever had sexual intercourse, good knowledge, and favourable attitudes were all determinant factors of SRH service utilization among in-school adolescents with disabilities. So, it is recommended that the Jimma zone administration, disability center organizations, and other governmental and NGOs should give attention to SRH services based on the type of disability and sex and work on creating awareness and a favourable attitude.

## Figures and Tables

**(a):** 

Sample size calculation for the prevalence of SRH service utilization						
Variables	Prevalence (*P*)	Confidence level	Margin error	Sample size	Design effect	Total sample size
SRH service utilization	26.1	95%	5%	294	1.5	444

**(b):** 

Sample size determined for factors affecting SRH service utilization								
Variable	Confidence level	Power	Unexposed : exposed	%among exposed	%among nonexposed	Sample size	Design effect	Total sample size
Disability type	95%	80%	1	32.7	6.2	84	1.5	127

**Table 2:** Sociodemographic characteristic of in-school adolescents with disability in Jimma zone, southwest Ethiopia, 2021 (*N* = 454).

Variables	Categories	Frequency	Percent
Sex of respondent	Male	258	56.8
	Female	196	43.2

Age (years)	10-14	194	42.7
	15-19	260	57.3

Religion	Muslim	320	70.5
	Protestant	36	7.9
	Orthodox	91	20
	Other	7	1.5

Educational status	1-4	219	48.2
	5-8	142	31.3
	9-12	93	20.5

Living condition	Both parents	262	57.7
	Either of the parents	113	24.9
	Partner	12	2.6
	Relative	53	11.7
	Guardian	8	1.8
	Alone	6	1.3

Source of income	Yes	46	10.1
	No	408	89.9

**Table 3:** Form and time of disability among in-school adolescents with disability of Jimma zone, southwest Ethiopia, 2021 (*N* = 454).

Variables	Frequency	Percentage
Form of disability		
Visual impairment	97	21.4
Hearing impairment	125	27.5
Physical impairment	88	19.4
Mental impairment	144	31.7
Severity of disability		
Partial impairment	270	59.5
Severe impairment	184	40.5
Time of disability		
From birth	242	53.3
Early childhood	152	33.5
Later in life	60	13.2

**Table 4:** Awareness and attitude toward sexual and reproductive health services among in-school adolescents with disability in Jimma zone, southwest Ethiopia, 2021.

Variables	Responses	Frequency	Percent
Know the type of SRH services	Family planning	265	49
	VCT	116	21.4
	STI diagnosis and treatment	86	15.9
	SRH information and education	42	7.8
	Postabortion care	32	5.9

Source of information about SRH services	Radio	78	22.0
	Television	114	32.1
	Health facility	36	14.1
	Peers	34	9.6
	Family	4	1.1
	School	85	23.9
	Other	4	1.1

Know where to get the SRH services	Yes	205	57.1
	No	154	42.9

Attitude towards SRH services	Unfavourable	230	50.7
	Favourable	224	49.3

Self-risk perception of contracting STIs including HIV/AIDS	High risk	70	15.4
	Low risk	282	62.1
	Not at all	102	22.5

Reason for high-risk perception	Multiple sexual partners	17	24.3
	Visited commercial sex	41	58.6
	Do not use a condom at all	6	8.6
	Do not use condom consistently	6	8.6

**Table 5:** Sexual and reproductive health service utilization among in-school adolescents with disability in Jimma zone, southwest Ethiopia, 2021.

Variables	Responses	No.	%
Sexual and RH service utilization	SRH information and education	38	8.4
	VCT	78	17.2
	STI diagnosis and treatment	26	5.7
	Contraceptive method use	26	5.7
	Others	11	2.4

The approach of SRH service providers	Attractive	37	47.4
	Not attractive	41	52.6

Visited YRHS but missed the service	Yes	24	31.6
	No	52	68.4

Need assistant to get SRH service	Yes	262	57.7
	No	192	42.3

The existing SRH service disability friendly	Yes	134	29.5
	No	320	70.5

Reason for not disability friendly	Inconvenient road	144	21.2
	Inconvenient service delivering	118	17.4
	Lack of comfort & privacy	128	18.9
	Distance from health facility	169	24.9
	Long waiting hour	120	17.7

Available health care facility physically accessible	Yes	68	15
	No	386	85

**Table 6:** Sexual and reproductive health status among in-school adolescents with disability in Jimma zone, southwest Ethiopia, 2021.

Variables		Frequency	Percentage
Had sexual intercourse	Yes	48	10.6
	No	406	89.4

Reason for sexual intercourse	Married	10	20.8
	It is natural	20	41.7
	Rape	2	4.2
	Peer pressure	14	29.2
	Drunk alcohol	2	4.2

Type of contraceptive method used	Pills	8	30.8
	Condom	2	7.7
	Implants	2	7.7
	Injections	12	46.2
	Post pills	2	7.7

History of pregnancy	Yes	4	14.3
	No	24	85.7

**Table 7:** Determinant factors of sexual and reproductive health service utilization among in-school adolescents with disability in Jimma zone, southwest Ethiopia, 2021.

Variables	SRH service utilization		COR (95% CI)	AOR (95% CI)
	Yes	No		
Form of disability				
Visual impairment	34 (7.5%)	63 (13.9%)	5.93 (2.88-12.24)	3.34 (1.33-8.42)^∗^
Hearing impairment	14 (3.1%)	111 (24.4%)	1.38 (0.61-3.12)	0.71 (0.26-1.94)
Physical impairment	32 (7%)	56 (12.3%)	6.28 (3.01-13.08)	9.98 (3.49-28.51)^∗∗^
Mental impairment	12 (2.6%)	132 (29.1%)	1	1
Time of disability				
From birth	54 (11.9%)	188 (41.4%)	0.67 (0.35-1.25)	1.95 (0.79-4.82)
Early childhood	20 (4.4%)	132 (29.1%)	0.35 (0.17-0.73)	0.67 (0.26-1.73)
Later in life	18 (4%)	42 (9.3%)	1	1
Sex				
Male	66 (14.5%)	192 (42.3%)	2.24 (1.36-3.70)	2.32 (1.18-4.57)^∗^
Female	26 (5.7%)	170 (37.4%)	1	1
Self-risk perception of STIs				
High risk	26 (5.7%)	44 (9.7%)	2.75 (1.36-5.57)	2.19 (0.84-5.72)
Low risk	48 (10.6%)	234 (51.5%)	0.96 (0.52-1.73)	0.71 (0.34–1.49)
Not at all	18 (4%)	84 (18.5%)	1	1
Had sexual intercourse				
Yes	20 (4.4%)	28 (6.2%)	3.31 (1.76-6.21)	5.34 (2.05–13.92)^∗∗^
No	72 (15.9%)	334 (73.6%)	1	1
Attitudes				
Unfavourable	30 (6.6%)	200 (44.1%)	1	1
Favourable	62 (13.7%)	162 (35.7%)	2.55 (1.57-4.13)	3.11 (1.59-6.07)^∗∗^
Educational status				
1-4	28 (6.2%)	191 (42.1%)	1	1
5-8	32 (7%)	110 (24.2%)	1.98 (1.14-3.47)	1.56 (0.76-3.18)
9-12	32 (7%)	61 (13.4%)	3.57 (1.99-6.41)	2.68 (1.11-6.47)^∗^
Age (years)				
10-14	48 (10.6%)	146 (32.2%)	1.61 (1.01-2.56)	3.84 (1.83-8.05)^∗∗^
15-19	44 (9.7%)	216 (47.6%)	1	1
Knowledge				
Good	46 (12.3%)	70 (18.7%)	3.04 (1.86-4.960)	2.74 (1.35-5.54)^∗^
Poor	46 (12.3%)	213 (56.8%)	1	1

^∗∗^ *P* < 0.001 and ^∗^*P* < 0.05. SRH: sexual and reproductive health; 1: reference category; COR: crude odds ratio; AOR: adjusted odds ratio; CI: confidence interval.

## Data Availability

Data will be available upon request from the corresponding author.

## Abbreviations

YPWD: Young people with disability
SRH: Sexual and reproductive health
VCT: Voluntary counselling and testing
STI: Sexual transmitted infections
AOR: Adjusted odds ratio
CI: Confidence interval
COR: Crude odds ratio.
